# Association of Sand Dust Particles with Pulmonary Function and Respiratory Symptoms in Adult Patients with Asthma in Western Japan Using Light Detection and Ranging: A Panel Study

**DOI:** 10.3390/ijerph121013038

**Published:** 2015-10-16

**Authors:** Masanari Watanabe, Hisashi Noma, Jun Kurai, Atsushi Shimizu, Hiroyuki Sano, Kazuhiro Kato, Masaaki Mikami, Yasuto Ueda, Toshiyuki Tatsukawa, Hideki Ohga, Akira Yamasaki, Tadashi Igishi, Hiroya Kitano, Eiji Shimizu

**Affiliations:** 1 Department of Respiratory Medicine and Rheumatology, Tottori University Faculty of Medicine, 36-1 Nishi-cho, Yonago 683-8504, Japan; E-Mails: junkurajun@gmail.com (J.K.); uedayasuto@med.tottori-u.ac.jp (Y.U.); yamasaki@med.tottori-u.ac.jp (A.Y.); igishi@med.tottori-u.ac.jp (T.I.); eiji@med.tottori-u.ac.jp (E.S.); 2 Department of Data Science, The Institute of Statistical Mathematics, 10-3 Midori-cho, Tachikawa, Tokyo 190-8562, Japan; E-Mail: noma@ism.ac.jp; 3 Regional Atmospheric Environment Section, Center for Regional Environmental Research, National Institute for Environmental Studies, 16-2, Onogawa, Tsukuba 305-8506, Japan; E-Mail: shimizua@nies.go.jp; 4 Department of Respiratory Medicine and Allergology, Kinki University Faculty of Medicine, 377-2 Ohnohigashi, Osakasayama 589-0014, Japan; E-Mail: hsano@med.kindai.ac.jp; 5 Department of Respiratory Medicine, San-in Rosai Hospital, 1-8-1 Kaikeshinden, Yonago 683-0002, Japan; E-Mail: kato@saninh.rofuku.go.jp; 6 Hosshoji Clinic, 286-4 Hossyouji, Nanbuchou, Saihaku 683-0351, Japan; E-Mail: mahayachi87109@gmail.com; 7 Department of Respiratory Medicine, Matsue City Hospital, 32-1 Noshirachou, Matsue 690-0045, Japan; E-Mail: mailtatsukawa@yahoo.co.jp; 8 Ohga Clinic, 4-2-1 Nishifukuhara, Yonago 683-0805, Japan; E-Mail: ohgayonago@gmail.com; 9 The Board of Directors, Tottori University, 36-1 Nishi-cho, Yonago 683-8504, Japan; E-Mail: hkitano@med.tottori-u.ac.jp

**Keywords:** adult asthma, light detection and ranging, peak expiratory flow, respiratory symptom, sand dust particles

## Abstract

Light detection and ranging (LIDAR) can estimate daily volumes of sand dust particles from the East Asian desert to Japan. The objective of this study was to investigate the relationship between sand dust particles and pulmonary function, and respiratory symptoms in adult patients with asthma. One hundred thirty-seven patients were included in the study. From March 2013 to May 2013, the patients measured their morning peak expiratory flow (PEF) and kept daily lower respiratory symptom diaries. A linear mixed model was used to estimate the correlation of the median daily levels of sand dust particles, symptoms scores, and PEF. A heavy sand dust day was defined as an hourly concentration of sand dust particles of >0.1 km^−1^. By this criterion, there were 8 heavy sand dust days during the study period. Elevated sand dust particles levels were significantly associated with the symptom score (0.04; 95% confidence interval (CI); 0.03, 0.05), and this increase persisted for 5 days. There was no significant association between PEF and heavy dust exposure (0.01 L/min; 95% CI, −0.62, 0.11). The present study found that sand dust particles were significantly associated with worsened lower respiratory tract symptoms in adult patients with asthma, but not with pulmonary function.

## 1. Introduction

Turbulent winds disperse large quantities of particulate desert matter, including dust and sand over large areas, and these compounds induce disease in humans [1] and impact the health of widespread populations over large distances as they travel far from their source [2]. Deserts in China and Central Asia produce the second largest volume of dust emissions worldwide, contributing to approximately 20% of the global total [3]. These sand dust storms, referred to as Asian dust storms (ADS), have travelled to the West coast of the United States [4,5] and occasionally around the globe [6].

Aeolian sand dust in ADS is comprised of silicon dioxide (SiO_2_) and aluminum oxide (Al_2_O_3_), with other significant components, including iron (III) oxide (Fe_2_O_3_), calcium oxide (CaO), and magnesium oxide (MgO) [7]. Sand dust emissions originating from China and Central Asia have recently become an even greater health concern because they now contain anthropogenic metals, chemicals, and microorganisms introduced by rapid industrial development [8,9]. A number of epidemiologic studies have measured the effects of ADS on human health [10], and have found increases in hospital admissions and the deterioration of respiratory symptoms and pulmonary function in patients with asthma [11,12,13,14]. A study by Park *et al.* that attempted to investigate the association of ADS with respiratory symptoms found that the levels of particulate matter smaller than 10 μm (PM_10_) during ADS periods were significantly associated with pulmonary function and night-time symptoms [15]. There is currently no uniform international definition of ADS. Thus, the actual impact of sand dust exposure during periods of ADS on pulmonary function and respiratory symptoms remains unclear, particularly in adult patients with asthma.

The diameter of articulate matter, specifically PM_10_ (<10 μm) and PM_2.5_ (<2.5 μm), has routinely been used to measure air quality, and most studies to date have assessed the relationship between sand dust storms and disease based on PM_10_ and PM_2.5_ values. However, PM_10_ and PM_2.5_ represent a complex mixture of solid and liquid particles of varying compositions. In contrast, light detection and ranging (LIDAR) depolarization provide air quality measurements by two wavelengths that are simultaneously applied within <1 km above ground [16,17]. The LIDAR system detects sand dust particles and aerosolized air pollutants using the extinction coefficient. Although LIDAR is unable to distinguish the size of particles, it can distinguish the shape of particles and measure the quantity of non-spherical dust particles (representing sand dust particles) and spherical particles (representing aerosolized air pollutants). LIDAR systems have recently been installed widely throughout East Asia to measure the quantity of sand dust particles as they travel over long distances [18].

As noted, previous studies have examined the effects of air quality according to PM_10_ and PM_2.5_ measurements on respiratory function (*i.e.*, PEF) and symptoms in adult patients with asthma [19]. As such, in this study based on LIDAR measurements of Asian sand dust, we chose to investigate on these same parameters in adult patients with asthma in Japan and examine their relationship with LIDAR values.

## 2. Experimental Section

### 2.1. Study Design

This longitudinal study was conducted in Western Japan from March 2013 to May 2013 by daily monitoring lower respiratory tract symptoms and morning PEF measurements in adult patients with asthma. One hundred fifty-one outpatients with asthma aged >18 years were recruited from December 2012 to January 2013. The patients resided within 25 km of Tottori University Hospital in Yonago City, which is located in Western Japan and includes four locations: Yonago City, Matsue City, Sakaiminato City, and Yasugi City. Patients were diagnosed with asthma based on the Global Initiative for Asthma (GINA) criteria and were included upon meeting criteria (1) and (2) or criterion (3) as follows: (1) a history of intermittent wheezing; (2) airway hyperresponsiveness to methacholine; and (3) reversible airflow limitation (12% and 200 mL variability in forced expiratory volume in 1-s (FEV_1_) [20]. Allergic rhinitis and/or chronic sinusitis were considered present based on diagnosis by an otolaryngologist. Atopic disposition was diagnosed based on the serum total immunoglobulin E levels divided by the standard value and/or on a positive result in a specific immunoglobulin E test using MAST33 conducted in February 2013. Pulmonary function tests were also performed in all patients in February 2013. This study was approved by the institutional ethics committee (Ethics Committee of Tottori University, approval No. 1656), and all patients provided written informed consent.

### 2.2. Definition of the Heavy Dust Days and Monitoring of Air Pollutants

Daily information on ADS events was obtained from the Japan Meteorological Agency. Concentrations of suspended particle matter (SPM), sulfur dioxide (SO_2_), nitrogen dioxide (NO_2_), and photochemical oxidants (O_x_) are monitored at multiple locations throughout Japan by the Japanese Ministry of the Environment. Data for the SPM, PM_2.5_, SO_2_, NO_2,_ and O_x_ collected in Yonago City were used in the present analysis. LIDAR data for sand dust particles and aerosolized air pollutants were obtained from the Matsue Observatory.

LIDAR systems measure aerosol levels at 15-min intervals by distinguishing between non-spherical particles and spherical particles [16,17,21]. This study used values measured from 120 to 150 m above ground, which is the minimum altitude required for LIDAR systems to measure non-spherical and spherical particles. Data recorded by LIDAR during low clouds, fog, or rain were excluded from further analysis. Daily particle levels were determined based on the median value of 96 measurements collected over a 24-h period from midnight of 1 day to midnight of the next day. The daily levels were only calculated when the number of available measurements exceeded 50% of the total number of measurements. The hourly value was defined as the mean of four measurements per hour. The hourly values were not calculated when the number of available measurements was less than two. Daily particle levels were analyzed to investigate the association of sand dust particles and aerosolized air pollutants with PEF. An hourly value of 0.1 km^−1^ is the lowest detectable value in real time, and the hourly value is presented on the Japanese Ministry of the Environment’s website [22]. A heavy dust day was defined as a day in which hourly sand dust particle values exceeded 0.1 km^−1^. Eight heavy dust days, 7–10 March, 17 March, 20 March, 6 April, and 30 April, occurred during the study period.

### 2.3. Recording the Daily Lower Respiratory Tract Symptoms and Morning PEF

From February 2013 to May 2013, with February serving as the practice period, all patients recorded their daily morning PEF using a peak flow meter (Mini-Wright, Harlow, England, American Thoracic Society scale). Patients were instructed to measure the PEF three times every morning, before taking their inhaled corticosteroids, β_2_-agonists, or oral drugs. Each patient recorded the best value of three attempts. Scores for lower respiratory tract symptoms included cough, sputum, dyspnea, and wheezing and ranged from 0 (no symptoms) to 3 (symptoms occurring for most of the day). Scores were recorded nightly in a symptom diary. The total score for each lower respiratory tract symptom was calculated by summing the daily scores.

### 2.4. Statistical Analysis

The associations among sand dust particles and SPM, and PM_2.5_ levels were assessed by linear regression analysis using SPSS software (Japanese version 20.0 for Windows; IBM SPSS Japan Inc., Tokyo, Japan). Linear mixed models accounting for the correlations among repeated measurements for each patient were adopted to estimate the effect of exposure to sand dust particles and aerosolized air pollutants detected by LIDAR and to other air pollutants (SPM and PM_2.5_) on the total symptom score and daily PEF measurements [23,24]. Daily median levels were analyzed to investigate the association of sand dust particles and aerosolized air pollutants with PEF. The daily (24-h) average levels of air pollutants (SPM, PM_2.5_, SO_2_, NO_2_, and O_x_) and meteorological variables (daily temperature, humidity, and atmospheric pressure) were used in the analysis. The linear mixed models included a random intercept for patients in the analysis and individual factors, including age, sex, smoking status, the presence of allergic rhinitis and atopic disposition, treatment step, pulmonary function, levels of air pollutants (SO_2_, NO_2_, and O_x_), and meteorological variables (temperature, humidity, and atmospheric pressure). In the analysis using linear mixed models, the regression coefficient estimates were interpreted as the difference in the total symptoms score and PEF, and the 95% confidence intervals (CIs) were also obtained. For continuous air pollutant measurements, the results are presented as differences in outcomes per change in the interquartile range (IQR). The effect of heavy dust exposure and the post-exposure effect on the total symptom score and PEF were compared to those of non-heavy days over a lag period (lag) of 0–5 days after heavy dust exposure because in previous research the effect of heavy dust exposure on PEF has been found to persist for up 3 days [15]. For treating missing data, we used the multiple imputation method, which adequately addresses uncertainty of the imputed values based on multiply generated prediction values for missing data [25]. R, version 3.0.3 statistical software (R Foundation for Statistical Computing, Vienna, Austria) was used for all the statistical analyses. All *P* values are two-sided, and the significance level was set to 0.05.

## 3. Results

### 3.1. Patient Characteristics

**Figure 1:**
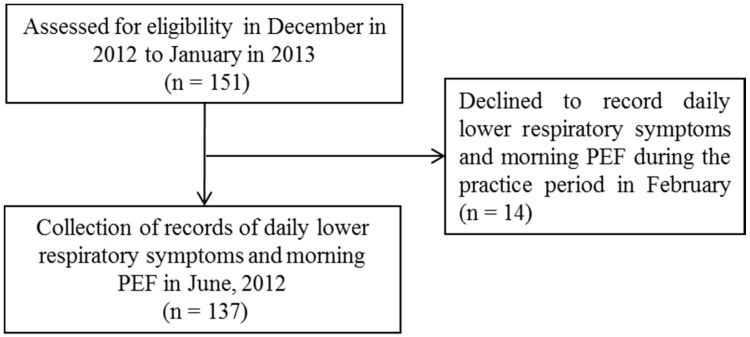
Patient selection flow chart, (PEF, peak expiratory flow).

A patient-selection flow chart is shown in Figure 1. Fourteen of 151 study patients did not complete daily symptom diaries or PEF measurements during the practice period (February) and were subsequently excluded from the analysis (Figure 1). The remaining 137 patients consistently recorded daily respiratory symptoms and the PEF for >90% of the study period (March to May), and these 137 patients were included in the final analysis. Their characteristics are shown in Table 1. According to the GINA criteria, the treatment step, which corresponded to the patient’s asthma control level, was used in March 2013 [26].

**Table 1:** Patients’ characteristics.

Characteristic	Mean/Number (%)
Number of patients	137
Age (years)	63.5 ± 15.4
Sex (male/female)	58/79
Smoking status	
Never	92 (67.1)
Former	38 (27.7)
Current	7 (5.2)
Pulmonary function	
FVC (L)	2.94 ± 0.70
FEV_1_ (L)	2.09 ± 0.60
%FEV_1_ (%)	100.4 ± 24.7
Allergic rhinitis and/or chronic sinusitis	60 (43.8)
Atopic disposition	74 (54.0)
Treatment step	
Step 1	1 (0.7)
Step 2	14 (10.2)
Step 3	29 (21.2)
Step 4	87 (63.5)
Step 5	6 (4.4)

Data are shown as the mean ± standard deviation or the number (percentage) of patients. FEV_1_: forced expiratory volume in 1 second; %FEV_1_: percentage of predicted FEV_1_; FVC: forced vital capacity. According to the Global Initiative for Asthma criteria, the treatment step, which corresponded to the patient’s asthma control level, was used in March 2013.

### 3.2. Sand Dust Particle and Aerosolized Air Pollutant Levels

**Figure 2:**
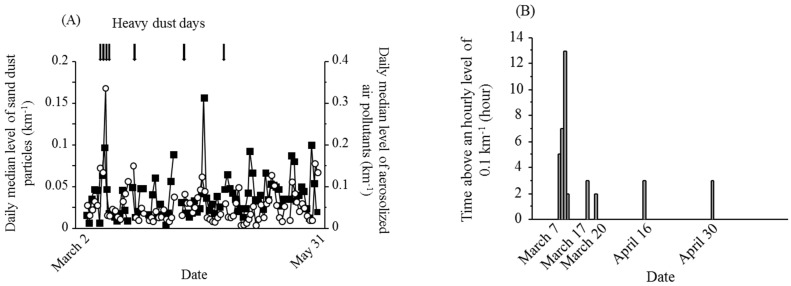
Sand dust particle and aerosolized air pollutant levels (**A**) Daily median levels of sand dust particles (open circles) and aerosolized air pollutants (closed squares). A heavy dust day was defined as an hourly sand dust particle level >0.1 km^−1^ using light detection and ranging data. Arrows indicate heavy dust days; (**B**) The time above 0.1 km^−1^ was based on hourly levels. Eight heavy dust days, 7–10 March, 17 March, 20 March, 6 April, and 30 April, occurred during the study period.

**Figure 3:**
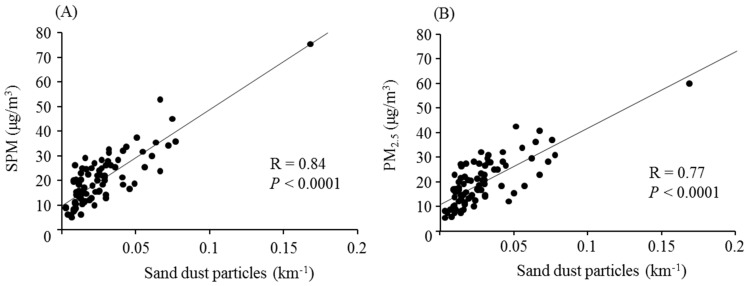
Associations of sand dust particles with SPM and PM_2.5_ Sand dust particles were significantly associated with suspended particulate matter (SPM) (**A**) and particulate matter <2.5 μm (PM2.5) (**B**).

The daily levels of sand dust particles and aerosolized air pollutants over the study period are shown in Figure 2A. The Daily levels were not calculated during 7 days of the study period (1, 19, and 25 March; 6, 7, and 24 April; and 19 May). The rate of missing 15-min measurement intervals from 1 March to 31 May was 11.8%. Figure 2B shows the mean time above 0.1/km^−1^ during the 8 heavy dust days. Sand dust particles were significantly associated with SPM and PM_2.5_ (Figure 3).

### 3.3. Lower Respiratory Symptoms and Peak Expiratory Flow

The estimated changes in the total lower respiratory tract symptom score and PEF per IQR increase in the levels of sand dust particles, aerosolized air pollutants, SPM, and PM_2.5_, after adjusting for individual patient characteristics, gaseous air pollutants, and meteorological variables are shown in Table 2. The total symptom score was significantly associated with sand dust particle, SPM, and PM_2.5_ levels. However, there was no significant association of PEF with the sand dust particle, aerosolized air pollutant, SPM, or PM_2.5_ levels.

**Table 2:** Associations of the lower respiratory tract symptom score and PEF to exposure to various environmental parameters.

	Meteorological Exposure			
	Sand Dust Particles	Aerosolized Air Pollutants	SPM	PM_2.5_
IQR	0.02 km^−1^	0.06 km^−1^	12.8 μg/m^3^	13.6 μg/m^3^
Change in the symptoms score	0.04	0.02	0.06	0.07
95% CI	0.03, 0.05	−0.01, 0.04	0.04, 0.08	0.04, 0.08
*P* value	<0.001	0.085	<0.001	<0.001
Change in the PEF (L/min)	0.01	−0.17	−0.13	−0.20
95% CI	−0.62, 0.11	−0.67, 0.33	−0.60, 0.35	−0.80, 0.41
*P* value	0.946	0.507	0.588	0.526

Associations to sand dust particles, aerosolized air pollutants, SPM, and PM_2.5_ were evaluated in a linear mixed-effects model after adjusting for individual characteristics, gaseous air pollutants, and meteorological variables. Daily median levels were analyzed to investigate the association of sand dust particles and aerosolized air pollutants with PEF. The daily (24-h) average levels of air pollutants (SPM, PM_2.5_, SO_2_, NO_2_, and O_x_) and meteorological variables (daily temperature, humidity, and atmospheric pressure) were used in the analysis. IQR, interquartile range; CI, confidence interval; NO_2_, nitrogen dioxide; O_x_, photochemical oxidants; PEF, peak expiratory flow; PM_2.5_, particulate matter < 2.5 μm in diameter; SO_2_, sulfur dioxide; SPM, suspended particulate matter.

The estimates for the associations of heavy dust days with a total lower respiratory tract symptom score and PEF after adjusting for individual characteristics, gaseous air pollutants, and meteorological variables are shown in Table 3. The total symptom score showed significant increase with exposure to heavy dust compared to non-heavy dust days. The effects of the heavy dust on the total symptom score were 0.15 on lag 0, 0.12 on lag 0–1, 0.11 on lags 0–2 and 0–3, 0.08 on lags 0–4, and 0.06 on lags 0–5. The greatest increase in the total symptoms score occurred on lag 0, and the significant associations persisted for 5 days. However, there were no significant differences in the PEF values between heavy and non-heavy dust days.

**Table 3:** Association of heavy dust exposure with the change in lower respiratory tract symptoms and the PEF.

**Lag Time (Days)**	**Change in the Symptom Score**	**95% CI**	***P* Value**
Lag 0	0.15	0.09, 0.20	<0.001
Lag 0–1	0.12	0.07, 0.16	<0.001
Lag 0–2	0.11	0.07, 0.15	<0.001
Lag 0–3	0.11	0.07, 0.14	<0.001
Lag 0–4	0.08	0.05, 0.12	<0.001
Lag 0–5	0.06	0.02, 0.09	0.003
**Lag Time (Days)**	**Change in the PEF (L/min)**	**95% CI**	***P* Value**
Lag 0	−0.18	−1.61, 1.25	0.805
Lag 0–1	−0.21	−1.42, 0.99	0.729
Lag 0–2	0.11	−0.95, 1.17	0.836
Lag 0–3	0.18	−0.82, 1.17	0.730
Lag 0–4	0.09	−0.86, 1.03	0.856
Lag 0–5	0.36	−0.56, 1.27	0.444

The change in the PEF and symptom score was adjusted for individual patient characteristics, gaseous air pollutants, and meteorological variables. A heavy dust day was defined as an hourly level of sand dust particles >0.1 km^−1^ using light detection and ranging data. The total score for each lower respiratory tract symptom, ranging from 0 to 3, was calculated by summing the scores for cough, sputum, dyspnea, and wheezing each day. PEF: peak expiratory flow, CI: confidence interval.

## 4. Discussion

A key issue in determining the association between sand dust exposure and health, particularly respiratory disease, is how best to measure the volume of airborne sand dust particles. The concentration of sand dust emissions has conventionally been expressed in terms of the PM_10_ and PM_2.5_. However, PM_10_ and PM_2.5_ measurements include a mixture of various components in addition to sand dust particles. LIDAR can quantitate the diffusion of sand dust particles from East Asia deserts to Japan by distinguishing between non-spherical dust particles and spherical dust particles. This current study used LIDAR data to examine the association of sand dust particles with pulmonary function (PEF) and lower respiratory tract symptoms in adult patients with asthma in Western Japan. Our key finding, based on the results from heavy sand days, was that sand dust particle exposure was significantly associated with worsened lower respiratory tract symptoms but not with measureable changes in PEF.

The finding that heavy sand days were not accompanied by significant changes in PEF, despite worsened respiratory symptoms, was expected. Maestrelli *et al.* have shown that PM_10_ exposure was associated with decreased asthma control and a worsened health-related quality of life, but they did not find a significant association between PM_10_ exposure and short-term changes in pulmonary function or inflammation [27]. Further, the inability to detect a decrease in PEF associated with sand dust particle exposure may be due to the lack of sensitivity of PEF for detecting subtle changes in asthma. Chang-Yeung *et al.* found that PEF monitoring is not as sensitive as a symptom diary for predicting asthma exacerbations [28]. Additionally, in the present study, most of the patients may have had well-controlled asthma due to proactive treatment based on the percentage of predicted FEV_1_. Thus, we did not find a significant association between pulmonary function and sand dust particles. Although several studies have shown that PM_10_ and PM_2.5_ levels are the most consistent factors related to pulmonary function in asthma [29,30], we may have been similarly unable to detect any significant associations of pulmonary function with PM_10_ and PM_2.5_ due to limited sensitivity. 

We found that ADS had a significant association with lower respiratory tract symptoms in asthmatic adults until 5 days after heavy dust exposure. Park *et al.* have reported that the effect of dust exposure on PEF persisted for up 3 days [15], and Kanatani *et al.* showed that the risk for hospitalization persisted for up to 1 week after heavy dust events [11]. Our results are comparable to these earlier findings and suggest that the influence of heavy dust exposure on adult patients with asthma may be prolonged.

There is currently no standardized international definition of heavy dust days caused by dust arising from the deserts of East Asia. Similarly, LIDAR systems lack defined criteria for heavy dust days. Previous studies using LIDAR data [11] have defined ADS events as daily (24-h) median sand dust particle levels of 0.066 km^−1^ (moderate ADS day) and 0.105 km^−1^ (heavy ADS day) [31], or >0.1 km^−1^. During our study period, the daily median level never exceeded 0.06 km^−1^. Furthermore, in Japan, the daily median sand dust particle level using LIDAR data is not widely available to the public. Instead, the website of the Japanese Ministry of the Environment shows the hourly level, which may be more useful to patients who are attempting to avoid the influence of heavy dust exposure on health. Therefore, in the present study, we defined a heavy dust day according to these hourly recordings as a sand dust particulate level of >0.1 km^−1^ within a 1-hour period because >0.1 km^−1^ is the lowest detectable value in real time. We observed a significant association between heavy dust exposure and lower respiratory tract symptoms when heavy dust days were defined as an hourly level >0.1 km^−1^.

Heavy days have been further defined by the Japan Meteorological Agency as days on which visibility is reduced to <10 km due to dust arising from the deserts of East Asia and detected by meteorological satellites. There were four heavy dust days (8–10, 19, and 20 March) based on this definition during our study period, and on these days we did find significant changes in the PEF (−1.76 L/min; 95% CI, −3.30, −0.21; *P* = 0.026) along with significant associations between lower respiratory symptoms and heavy dust. However, visibility is reduced by the presence of other air pollutants as well as sand dust particles, and we speculated that this may have accounted for these differences in the results.

ADS have been associated with a rise in hospital admissions along with worsening symptoms and pulmonary function in patients with asthma [11,12,13,14]. Two patients were hospitalized due to exacerbation of asthma during the present study, but these hospitalizations occurred more than 7 days after any heavy dust day. The lower hospitalization rate and lack of significant changes in pulmonary function during heavy dust exposure in our study may also have been related to levels of SPM and sand dust particles set by our criteria compared to those of previous studies. Regardless, the finding of an exacerbation of lower respiratory tract symptoms during heavy dust days in patients with asthma during our study period was consistent with the findings in Min *et al.*’s study [32], and the results support the assertion that exposure to sand dust particles contributes to the deterioration of lower respiratory tract symptoms in patients with asthma. According to the study by Higashi *et al.* from Japan, the prevalence of cough in subjects with asthma was below 25% during ADS [33]. In other words, very few patients with asthma experience deterioration of respiratory symptoms during ADS. Thus, the effect of ADS on respiratory symptoms in patients with asthma may be not severe in Japan.

Conventional PM_10_ and PM_2.5_ measurements are recorded at a lower altitude than LIDAR measurements of non-spherical and spherical particles. LIDAR systems are unable to accurately measure these particles below an altitude of 120 m. Thus, along with Ueda *et al.* and Kanatani *et al.* [11,13,20], this study used LIDAR data measured from 120–150 m above ground, although these altitudes are not optimal for estimating potential dust exposure for humans. While the result of this study suggests that LIDAR systems can be suitable for studying the association of dust emissions from East Asian deserts and health, the LIDAR systems were unable to definitively identify a heavy dust event; thus, additional studies are needed to address this.

We did not find a significant association between aerosolized air pollutants and pulmonary function and lower respiratory tract symptoms in this study. Spherical particles (aerosolized air pollutants) consist of various substances such as sulfate, nitrate, or organic carbon, and the hygroscopicity of spherical particles is much greater than that of non-spherical particles (*i.e.*, sand dust particles). Therefore, the higher the humidity, the bigger the spherical particles become. PM_2.5_ measurements at ground level are adjusted for humidity, but LIDAR systems are unable to adjust for humidity. As a result, the level of aerosolized air pollutants measured by LIDAR systems will ostensibly increase. LIDAR measurements of the actual quantity of air pollutants may be inconsistent, depending on humidity, and this may be why we were unable to find an association between aerosolized air pollutants and pulmonary function and lower respiratory tract symptoms.

The classification of the treatment step in patients with asthma is a reflection of asthma control. The classification of asthma severity is a poor indicator for required treatment and for what a patient’s response to that treatment may be [26]. An assessment of asthma control is more relevant and useful for guiding treatment [26,34]. The assessment of asthma control depends on symptom control and the future risk of adverse outcomes. Therefore, the treatment step reflects asthma control. Additionally, pulmonary function, particularly forced expiratory volume in 1 second (FEV_1_) as a percentage of predicted, is an important part of assessing the future risk. Age, sex, and allergic rhinitis are associated with the phenotype of severe asthma [35,36], and smoking and allergic rhinitis are risk factors for the exacerbation of asthma [35]. There is a relationship between atopic disposition and airway hyperresponsiveness [37]. Thus, the influence of air pollutants on asthma may differ with individual characteristics such as age, sex, smoking, the presence of allergic rhinitis and atopic disposition, treatment step, and pulmonary function. Therefore, we also adjusted for these individual characteristics in the analysis of the associations of air pollutants with PEF and lower respiratory tract symptoms. Several sensitivity analyses were performed; there were no differences in the results.

On certain days during the study period, the hourly sand dust particle level exceeded 0.1 km^−1^, although the daily levels were lower than during days with hourly levels <0.1 km^−1^. When the duration of the period in which particle levels exceed an hourly level of 0.1 km^−1^, which is brief, the daily level will remain relatively low. In addition, rain remarkably decreases the levels of sand dust particles and aerosolized air pollutants. Differences in the hourly and daily levels of sand dust particles during our study period were attributed to a brief duration of high-concentration periods and the occurrence of rain events.

There are several limitations to the present study. First, the interpretation was limited by a low incidence of only eight heavy dust days during the study period. Second, we were unable to estimate the amount of exposure to sand dust particles, aerosolized air pollutants, SPM, and PM_2.5_ in each individual patient and instead used measurements for locality. Third, we did not assess the amount of pollen, which may also affect pulmonary function. Fourth, we were also unable to investigate the effects of sand dust particles on airway inflammation. Exposure to particulate matter induces airway inflammation in asthma, which is characterized by increased numbers of neutrophils and elevated interleukin-8 and myeloperoxidase levels [38,39,40,41]. If the effects of sand dust particles, aerosolized air pollutants, SPM, and PM_2.5_ on pulmonary function and airway inflammation are studied at the same time, the association with sand dust emission in East Asia and respiratory health would be demonstrated more clearly. Fifth, asthma is a common disease in a wide range of age groups, and the patient population in our study was comprised of a large percentage of elderly patients. Sixth, to investigate the association with air pollution and asthma, numerous studies as well as randomized control studies on asthma have widely used symptom scores in conjunction with estimates of pulmonary function, but they lack validation and are interpreted inconsistently and uncritically. Similarly, symptom scoring in this study was not established, and at present, there is no established method of measuring daily asthma symptoms. However, in this study, the increase of symptom scores after heavy dust exposure had a clear time trend. Therefore, we suspect that exposure to sand dust particle can aggravate asthma symptoms. Finally, we only investigated the acute effects of the sand events on pulmonary function and lower respiratory tract symptoms. These limitations may over- or underestimate the effects of sand dust emissions in East Asia on pulmonary function and lower respiratory tract symptoms. To completely elucidate the associations of sand dust emission with pulmonary function and lower respiratory tract symptoms, we would have had to lengthen the study, and consequently the number of heavy dust days would have increased. We would then also monitor pollen levels and the effects of sand dust particles on airway inflammation. In addition, we would include younger patients to lower the mean age of the study.

## 5. Conclusions

Despite the limitations, this study has supported the suggestion that exposure to sand dust particles is significantly associated with worsened lower respiratory tract symptoms in adult patients with asthma. Patients and physicians should be adequately informed of the health implications of sand dust exposure to reduce these deleterious effects. LIDAR may be a useful for predicting potentially deleterious sand dust emissions on a day-to-day basis.

## Supplementary Files

Supplementary File 1
