# Supplementary material for: Association of Sand Dust Particles with Pulmonary Function and Respiratory Symptoms in Adult Patients with Asthma in Western Japan Using Light Detection and Ranging: A Panel Study
Source: Int J Environ Res Public Health. 2015 Oct 16;12(10):13038–52. doi: 10.3390/ijerph121013038 (PMC4627015; doi:10.3390/ijerph121013038)
Supplement: Supplementary File 1 [file ijerph-12-13038-s001.pdf]

Supplementary Information

Association of Sand Dust Particles with Pulmonary Function and Respiratory Symptoms in Adult Patients with Asthma in Western Japan Using Light Detection and Ranging: A Panel Study

Results

Sand Dust Particle and Aerosolized Air Pollutant Levels

Supplementary Figure S1 shows the total number of daily observable hourly levels. Light detection and ranging (LIDAR) was unable to calculate any hourly levels on March 25 and May 19. In the following analyses, these missing values were adequately treated using the multiple imputation method.

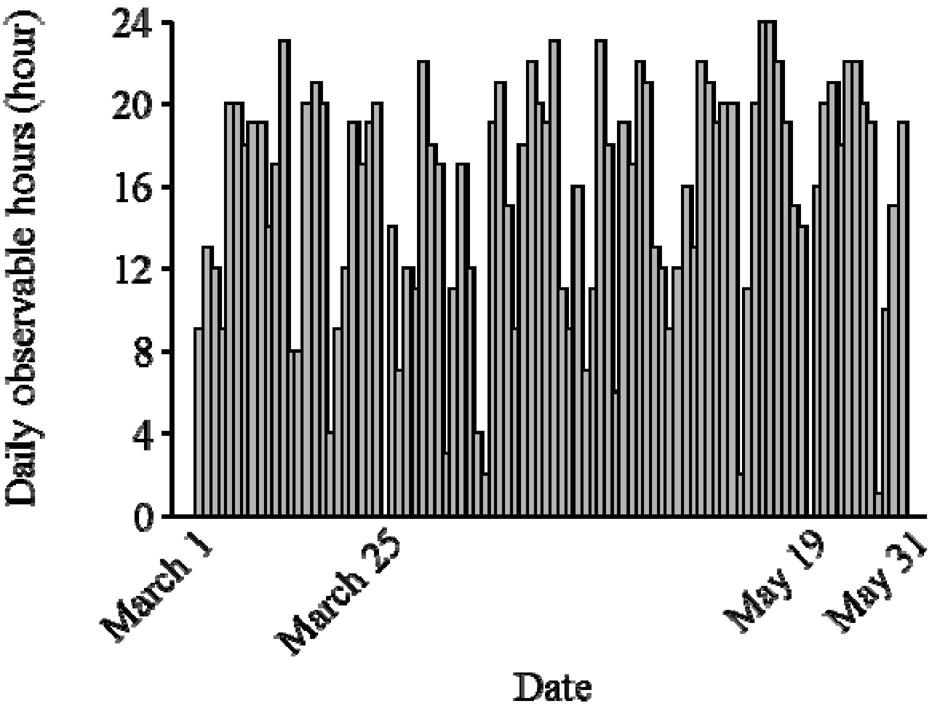

Figure S1. The total number of daily observable hours of the hourly level Light detection and ranging systems measure the aerosol levels at 15-min intervals. The hourly value is defined as the mean of four measurements per hour. If the number of measurements was less than two, the hourly values were not calculated.

Lower Respiratory Symptoms and Peak Expiratory Flow

The estimate of the changes in the total lower respiratory tract symptom score and peak expiratory flow (PEF) per interquartile range increased in the levels of SO_2, NO_2, and O_x, after adjusting for individual patient characteristics and the meteorological variables, as shown in Supplementary Table S1. The total symptom score was significantly associated with SO_2, NO_2, and O_x levels. However, there was no significant association of PEF with SO_2, NO_2, and O_x levels.

Table S1. Associations of the lower respiratory tract symptom score and PEF to exposure to SO_2, NO_2, and O_x.

	Meteorological Exposure		
	SO_2	NO_2	O_x
IQR	0.75 ppb	3.68 ppb	12.2 ppb
Change in the symptoms score	0.04	0.04	0.04
95% CI	0.02, 0.05	0.02, 0.06	0.01, 0.06
P value	<0.001	<0.001	0.001
Change in the PEF (L/min)	−0.01	0.11	−0.11
95% CI	−0.38, 0.35	−0.39, 0.62	−0.64, 0.44
P value	0.939	0.654	0.702

Associations to SO_2, NO_2, and O_x were evaluated in a linear mixed-effects model after adjusting for individual characteristics, gaseous air pollutants, and meteorological variables. The daily (24-h) average levels of air pollutants (SO_2, NO_2, and O_x) and meteorological variables (daily temperature, humidity, and atmospheric pressure) were used in the analysis.

IQR, interquartile range; CI, confidence interval; NO_2, nitrogen dioxide; O_x, photochemical oxidants; PEF, peak expiratory flow; SO_2, sulfur dioxide.

© 2015 by the authors; licensee MDPI, Basel, Switzerland. This article is an open access article distributed under the terms and conditions of the Creative Commons Attribution license (http://creativecommons.org/licenses/by/4.0/).
